# Enabling people, not completing tasks: patient perspectives on relationships and staff morale in mental health wards in England

**DOI:** 10.1186/s12888-015-0690-8

**Published:** 2015-12-02

**Authors:** Himanshu Mistry, William M. M. Levack, Sonia Johnson

**Affiliations:** 1 Division of Psychiatry, University College London, Maple House 149 Tottenham Court Road, W1T 7BN London, UK; 2 Hastings Community Mental Health Team, Hawke’s Bay District Health Board, Hastings, New Zealand; 3 Rehabilitation Teaching & Research Unit, University of Otago, Wellington, New Zealand

**Keywords:** Patient-staff relationships, Staff morale, Inpatient care, Consumer perspectives, Person-centredness, Qualitative research

## Abstract

**Background:**

Mental health inpatient wards are stressful places to work and concerns have been raised regarding quality of patient care and staff wellbeing on these wards. Recent research has suggested that robust support systems and conditions that allow staff to exercise professional autonomy in their clinical work result in better staff morale. Staff value having a voice in their organisations, and say that they would like more interaction with patients and processes to reduce violent incidents on wards. There has been little research into patients’ views on staff morale and on how it may impact on their care. This study aimed to explore staff morale and staff-patient relationships from a patient perspective.

**Methods:**

A qualitative investigation was conducted using purposive sampling to select seven inpatient wards in England representing various subspecialties. Semi-structured interviews were carried out with three patients on each ward. A thematic approach to analysis was used, supported by NVivo 10 software.

**Results:**

Patients valued staff who worked together as a cohesive team, treated them as individuals, practised in a collaborative way and used enabling approaches to support their recovery. Participating patients described observing staff closely and feeling concerned at times about their well-being and the impact on them of stress and adverse incidents. They tended to perceive ward staff and patients as closely and reciprocally linked, with staff morale having a significant impact on patient well-being and vice versa. Some participants also described modifying their own behaviour because of concerns about staff well-being. Administrative duties, staff shortages and detrimental effects of violent incidents on the ward were seen as compromising staff members’ ability to be involved with patients’ lives and care.

**Conclusion:**

Patient views about the factors impacting on staff morale on inpatient wards are similar to those of staff in qualitative studies. Their accounts suggest that staff and patient morale should be seen as interlinked, suggesting there is scope for interventions to benefit both.

## Background

Several investigations have raised concerns about the quality of care provided in inpatient mental health wards in the UK’s National Health Service (NHS) [1–3]. High staff vacancy and sickness rates, poor communication between community and inpatient teams, lack of leadership from consultant psychiatrists and limited availability of psychological treatments have all been identified as problems [4]. Mental health inpatient wards are potentially stressful places to work as patients tend to be very unwell, are often admitted against their will, and remain on wards for only short periods [5]. Staff on these wards are also frequently exposed to violence, further contributing to the stressful nature of the job [6].

Compounding this appears to be a discrepancy between what patients want from staff and what staff tend to deliver in their work roles. Hall has suggested that current nursing practice is often dominated by ‘social control functions’ such as observation, surveillance and management of difficult behaviours [7]. In contrast to this, developing a strong therapeutic relationship is associated with better patient experiences and satisfaction in inpatient settings [8, 9]. Patients place high value on staff members’ empathic qualities such as being caring, interested, understanding, respectful, and attentive. Indeed, the physical environment on inpatient wards, ward routines and the content of care are reported by patients to be less important than relationships with staff as priorities for improving their experiences of hospitalisation [10]. Initiatives reflecting these concerns have included the Productive Ward initiative, launched by the National Health Service Institute for Innovation and Improvement in 2008 [11]. It aimed to reduce activities that did not add value and focused on improving processes in inpatient settings in order to increase the amount of time that nursing and therapy staff spent with patients on therapeutic activities. This initiative has now been widely adopted by acute mental health services across the NHS, as has the Star Wards programme, which aims to promote “caring conversations” and enrich the range of activities in which staff and patients can engage together on wards [12] Concurrently, attention has increasingly been focused on how better to support mental health staff and reduce their levels of stress and burnout. In addition to a moral imperative to create healthy workplaces for staff members, greater staff wellbeing is likely to correlate with more positive patient outcomes, although evidence on this from mental health settings is very limited [13]. To investigate staff morale in inpatient settings and its potential determinants, Johnson and colleagues conducted the Inpatient Staff Morale Study – a mixed methods investigation of the factors contributing to high and low staff morale within inpatient mental health wards in the UK [14, 15]. This programme of research had three linked components. The first component was a large multicentre survey examining antecedents of high and low staff morale in 100 inpatient units nationally [14]. In the second component, a qualitative investigation of staff experiences and perspectives was undertaken, exploring the mechanisms by which high and low staff morale may develop [15]. Finally, in the third component (the study presented in this paper), a qualitative investigation was undertaken of inpatients’ perceptions of staff morale within inpatient mental health teams and of its impact on patients.

This third component of the study provides further triangulation regarding influences on staff morale, and also explores effects of good or poor staff morale on patients. In the first component of the study, results from the multicentre survey indicated that although inpatient mental health staff in the UK were generally satisfied with their work, a substantial number of people experienced ‘burnout’ in the workplace. In line with Karesek’s model of demand-support-control [16] this study indicated that burnout within mental health services tended to be associated with high work demands in combination with poor support systems and lack of professional autonomy [14]. Other factors contributing to low staff morale were lack of communication within teams, lack of role clarity, experiences of bullying, and violence.

In the second component of the study, results from the qualitative study of staff experiences suggested that low staffing levels were a particular concern on wards with low morale, with immediate impacts reported on workloads, training opportunities, supervision, exposure to risk, sickness absenteeism, and use of temporary ‘bank staff’ [15]. Front line staff also reported that lack of professional autonomy and limited opportunities to contribute to ward decisions could negatively affect morale, confirming quantitative results on the importance of autonomy [14]. Good teamwork and relationships with colleagues were conversely reported to be positive influences on staff morale. Consistent ward processes and policies and attention to role clarity were highlighted as necessary components of well-organised services. Staff also reported higher satisfaction when they were able to spend time with patients either for relationship building or therapeutic activities. In terms of the physical environment, good lighting and having space for group and individual activities and therapy were considered more important by staff than simply having attractive and comfortable work facilities.

The third component of the Inpatient Staff Morale Study, described in this paper, was deemed valuable as patients are very well placed to observe how mental health teams function and whether and how staff well-being and attitudes to work impact on patient care. Links between staff well-being and the experiences of patients however appear to have been explored relatively little in the mental health field. For example, a few quantitative findings have reported links between staff burnout and patient satisfaction [17], and between staff burnout and their own ratings of quality of care delivered to patients [18]. However, following searches with key text words and indexed subject headings in MEDLINE, CINAHL, and PsychINFO and searching of reference lists of relevant papers, we have not found any previous qualitative investigations regarding patients’ views about staff well-being, its antecedents and its impact on them.

### Aims

The aim of the study was to examine patients’ views on factors that contribute to good or poor staff morale on inpatient wards and on the impact of staff morale on patient experiences. Linked to this, the study also aimed to examine patients’ views on the staff-related factors that contribute to good clinical relationships and healthy ward environments. The over-arching purpose was to add to understanding of the mechanisms underlying good and poor staff morale, and to explore scope for improving both staff and patient well-being through staff-focused interventions.

## Methods

This study is the third component of a national multicentre study of staff morale in inpatient mental health wards which was commissioned by the National Institute of Health Research Service Delivery and Organisation Programme. The overall design was a sequential explanatory mixed methods study [19]: a large-scale quantitative study formed the first phase of the study, with data subsequently collected from staff and from inpatients on selected wards with the aim of further extending and allowing interpretation of quantitative findings on factors influencing morale. Wards were selected for the qualitative studies as scoring among the highest or lowest groups for morale in the initial survey. Multicentre ethics approval was obtained from the Hertfordshire Local Research Ethics Committee and site specific approvals from the participating Trusts.

### Setting

This qualitative investigation of patient views regarding staff morale and its impact on patient experiences was conducted on seven wards in London and the Midlands. These wards were selected from the 100 inpatient mental health wards across England that participated in the initial national survey on staff morale [13]. Based on survey data, purposive sampling was used to choose three wards in bottom quartile and four wards in top quartile for average staff morale scores. A further aim in the purposive sampling was to select wards that reflected various subspecialties (specifically, general acute wards, rehabilitation wards, a child and adolescent unit and a psychiatric intensive care unit).

### Participant recruitment

Participants were eligible for inclusion in this study if they were patients on the selected wards and being treated either voluntarily or under the Mental Health Act. All participants needed to be able to provide informed consent to participate in the study. Ward staff made an initial approach to potential participants whom they deemed able to understand the study and their potential role in it: they passed on details of potentially willing patients who were willing to be contacted and appeared to them to have capacity to consent to participating to study research staff. The study research workers, who had received specific training in assessing capacity and eliciting informed consent, then obtained signed written consent when appropriate. Patients were purposively selected to represent a range of age groups, ethnicities, diagnoses and lengths of stay. Three patients were selected from each of the seven wards involved in order to provide a range of viewpoints on different types of ward settings and from both high and low morale wards, resulting in a total sample of 21 interview participants. Their views regarding care received were unknown to researchers: thus selection on this basis was not feasible.

### Data collection

Written informed consent was obtained from all participants prior to data collection. Interviews with patients explored their experiences of the ward environment and their interactions with staff, and their views about staff morale and well-being. Interviews were conducted using a semi-structured, open-ended format by trained researchers. All interviews were conducted during the participants’ hospital admission and on the ward where they were residing. Each interview lasted approximately 30–45 min. The main questions focused on the patients’ experience of contact with staff, and on their impressions of staff experiences of work and life on the ward. Patients were asked specifically about their views about staff morale and how this might impact on patient care. Questions also explored the patients' views on existing staff practices and improvements which could be made to these.

### Data analysis

All interviews were recorded and transcribed verbatim. Data were examined using thematic analysis and constant comparative methods [20, 21]. Analysis focused on seeking answers to the initial research questions as well as allowing the emergence of unexpected ideas. Coding of transcripts was undertaken by one researcher (HM) with verification by a second (WL) to identify concepts and themes emerging from the interviews. Both of these researchers were blinded to ward morale categorisation for this initial analysis. Each transcript was read and re-read a number of times to compare emergent concepts and themes both across and within individual interviews. Following this initial analysis, ward morale categorisation was added to the coding of the interview transcripts in order to examine relationships between emerging themes and ward morale. As the analysis progressed, relationships between codes were identified so as to develop high order concepts. NVivo 10 software was used to help with management and manipulation of data.

To improve the credibility and trustworthiness of the analysis, initial coding of a few interviews was first undertaken independently by two researchers (HM and WL). These two researchers then compared their findings before progressing on with further data coding. As analysis progressed, the emerging results were discussed and debated with the whole research team. Negative case analyses—the purposeful exploration of ‘instances that do not fit the emerging' [22, p.174].—were used to further test and explore the emerging findings. The results below are presented alongside extracts from the participant interviews to illustrate key issues arising from the data.

## Results

### Participant characteristics

A summary overview of the participants’ characteristics is presented in Table 1 below. As intended, a range of demographic characteristics, ward types and morale levels were represented.

**Table 1:** Participant characteristics

	Participants from wards with high staff morale^a^ (*n* = 12)	Participants from wards with low staff morale^a^ (*n* = 9)
Gender	50 % (6/12) male; 50 % (6/12) female	89 % (8/9) male; 11 % (1/9) female
Age	25 % (3/12) 16–17 years	22 % (2/9) 18–25 years
	8 % (1/12) 18–25 years	33 % (3/9) 26–35 years
	25 % (3/12) 26–35 years	11 % (1/9) 36–45 years
	16 % (2/12) 36–45 years	33 % (3/9) 46–55 years
	8 % (1/12) 46–55 years	
	16 % (2/12) 56–65 years	
Ethnicity	50 % (6/12) British or White British	33 % (3/9) British or White British
	17 % (2/12) White & Black Caribbean	22 % (2/9) Black African
	8 % (1/12) Indian	11 % (1/9) Caribbean
	8 % (1/12) White other	11 % (1/9) Black other
	8 % (1/12) Black other	11 % (1/9) Asian
	8 % (1/12) Mixed ethnicity	11 % (1/9) Mixed ethnicity
Ward type	25 % (3/12) Female acute ward	33 % (3/9) Male psychiatric intensive care unit
	25 % (3/12) Mixed gender recovery unit	33 % (3/9) Male acute ward
	25 % (3/12) Mixed gender child and adolescent unit	33 % (3/9) Mixed gender acute ward
	25 % (3/12) Male rehabilitation unit	

^a^Wards were deemed to have ‘high’ or ‘low’ staff morale if they were in the top quartile or bottom quartile respectively for average staff morale scores in a survey of 100 inpatient mental health wards across England [13]

### Overview of findings

The central theme that emerged from these data was that of “enabling people; not completing tasks”. The patients viewed staff job satisfaction as largely arising from positive experiences of contributing to the lives of the patients. In this context staff morale could be high if opportunities existed for staff to interact positively on a personal level with patients and if teamwork and the work environment supported these endeavours. Conversely, staff morale was viewed by the patients as being under threat if barriers existed to meaningful staff-patient interactions (e.g. low staffing levels, administrative responsibilities which took staff away from the wards to offices) or if staff-patient interactions excessively depleted the staff members’ capacity to give of themselves (e.g. violent or abusive interactions that, in excess, became draining for staff). This central theme is described in relation to three main subthemes: A. The importance of collaborative interpersonal relationships between patients and staff, B. The importance of supportive teamwork, and C. Barriers to positive interpersonal relationships. A cross-cutting theme was the reciprocal nature of the staff-patient relationship, with patient and staff morale perceived as inter-dependent.

Of note, no clearly discernible differences were observed between patients who were on wards found in the initial quantitative survey to have high staff morale versus wards found to have low staff morale, either in terms the patients’ experiences of ward life, their attitudes towards staff, or their views on what constituted good or bad patient-staff interactions. Greater diversity of opinions was reported by participants within wards clustered by ‘high’ or ‘low’ morale than between ‘high’ and ‘low’ morale wards. Thus, in presentation of these results, the data from participants on both high and low morale wards have been combined.

A. The importance of collaborative interpersonal relationships between patients and staff

Patients placed high importance on having positive interpersonal relationships with staff and preferred staff who worked with them collaboratively. Staff morale and their ability to form positive relationships were often seen as closely and reciprocally linked. In terms of the kinds of relationships that patients wanted from staff, these could be categorized as: a) instrumental relationships, where staff provided a primarily service role (e.g. provision of medication and meals; provision of resources for activities; access to the outside world) and b) interpersonal relationships, where staff engaged with patients at an individually meaningful level (e.g. treating patients as people; treating them with dignity and respect; taking a collaborative approach to treatment, with there being a reciprocity of care – i.e. patients having opportunities to contribute to the lives of staff as well). While some patients sought only instrumental relationships with staff (these patients generally exhibiting very little interest in staff experiences of work life in the interviews) for the most part patients sought interpersonal relationships in the ward environment. This was reflected in concern for staff well-being as well as their own:

*I’d pick it up [staff stress] in a compassionate way and say look, you know, are you okay or something like that. I would care for them just as they were caring for me… You know I’m not that selfish as to think that everyone’s got to come and be happy-clappy people.*

*(Male, 46-55 years, Low morale acute ward)*

*There’s been couple of times that staff look down. I’ve said to them look is everything ok? They say it’s all right, don’t worry; I’m just having one of those off days. I don’t want it to be like that. I think oh my God what’s wrong, you know, have I done something or someone else done something? So I approach them and they just go, no, we’re okay.*

*(Male, 46 – 55 years, High morale Rehabilitation unit)*

*You think well, they do look tired so I am not going to ask and I just wait. I should put myself first, but if they look after us we look after them.*

*(Male, 36-45 years, Low Morale, Psychiatric intensive care unit)*

*Well I understand the stress of their job so I don’t demand too much and I will wait till an opportunity comes where I say, look you know, this and that, could you help me out? So I am quite sensitive to them which is good for them because that gives them space to be a bit more relaxed and do their job.*

*(Male, 46 – 55 years, Low morale, Acute inpatient ward)*

Some patients identified ways in which they were able to contribute to the running of the ward or to take on tasks related to their own recovery that might otherwise have been carried out by staff. This could be in the form of representation on ward community meetings to provide feedback to the managers regarding patient needs or complaints, or tasks such as organising activities for other patients, especially on the rehabilitation wards. Regaining independence was seen by patients as key to their recovery: thus staff support in enabling this was valued.

*Patient - Well, I tend to… They let me do quite a lot by myself. I go and see the accountant by myself. And I see the welfare officer and I also see the banker… so I’ve been sorting out a new flat and benefits and things like that.*

*Researcher - So they give you your independence?*

*Patient - They get me to stand for myself.*

(Female, 26–35 years, Low morale acute ward)

*Yes, I organise, like, game nights, bingo night and things like that, so I was just talking to one of the staff this morning about getting together and talking about having, like, organising some more activities, maybe, like a quiz night, or film night. They really help with activities on the ward and the ward manager gives them money to buy stuff for the bingo night and that, so everyone gets to win a prize.*

*(Male, 26–35 years, High morale rehabilitation unit)*

Patients also valued active involvement of staff in their life on the wards, with this involvement coming in many forms: one-to-one contact, participation in therapeutic activities, or joining them in their activities of daily living. The participants appreciated enthusiastic staff who took the initiative to be actively engaged in activities with patients.

*Patient - Because they don’t make it an ‘us’ and ‘them’. They make it an ‘our’. They come and join in with us in games and at lunch times and breakfast, the medication, they come out with medication and they join in with us. They even pour us teas and coffees…*

*Researcher - So there isn’t, you know, there isn’t a big split between patients and staff?*

*Patient - No, nothing like that at all.*

*(Female, 26–35 years, Low morale acute ward)*

One-to-one contact was, in particular, described as an important therapeutic resource for patients. The participants expressed appreciation for staff who tried their best to get one-to-one time with them despite other duties they needed to perform on the ward.

*She will sit down and spend time with you and talk to you and things like that. And I think that's all part of the treatment as well. I think, to be unwell and get the support that you need, and understanding what you need, does help, in a way, you know, because you know you’ve got people you can talk to and turn to and that, you know, and I think that's a big thing for people, you know, who do suffer from mental illness. Just having that bit of support and the understanding, you know.*

*(Male, 26–35 years, High morale rehabilitation ward)*

In addition to therapeutic contact, the patients also valued interactions with staff which were on friendly and informal terms. This seemed to provide an opportunity to the staff to get to know the patients as ‘people’.

*Well during protected time, we get to play games with each other, like, dominoes or scrabble, so we get to have a kind of fun interaction, a playful interaction.*

*(Male, 26–35 years, Low morale acute ward)*

Patients highly valued staff with excellent communication and listening skills; those who could identify a difficult situation early and address it with tact, allowing the patients to verbalise their feelings. Other staff were criticised for applying restraint and rapid tranquilisation methods without using de-escalation techniques first. Creating space for communication during a crisis situation was highly appreciated by the patients.

*When you’re stressed, they don’t just give you medication. They sit down and they talk to you and they only give you medication if you really need it. They don’t just go ‘here’s a pill’ and leave you alone. They’ll actually sit and listen, have a drink with you and calm you down and they’ll do techniques with you, like watch telly or talk, or read books and different distraction techniques and they teach you how to do that, which is a good thing.*

*(Female, 26–35 years, High morale acute ward)*

Regarding staff work satisfaction and well-being, participants expressed the view that providing good care and helping people when at their most vulnerable was potentially a great source of satisfaction and sense of achievement:

*I think it gives them something to look back at and think ‘I’ve helped him with this and now he’s on the right track’ sort of thing, you know.*

*(Male, 18–25 years, High morale recovery unit)*

B. Supportive teamwork enabling quality care

Good team work among staff was seen by patients as important in creating an atmosphere of security and containment in the ward environment. Patients felt able to identify teams that worked well and could be relied on for safe and efficient care. Efficient teams were viewed as both promoting good patient care and preserving staff morale. When teams did not function well, patients felt that staff attitudes could become unprofessional (such as by not attending to patient requests or to untoward events in a timely manner, or by making their views regarding the team known to the patients). Patients described a crucial role for leadership and management in promoting and maintaining good team work and in establishing clear and effective routines and procedures on the wards.

*I’ve never seen them argue in front of us, and they all seem to know what they’re doing, they’re all happy, they laugh, they talk. You know you notice when there’s banter going on.*

*(Male, 46–55 years, High morale rehabilitation ward)*

*The ward is managed very well. It’s got a routine and it is managed in an order that’s good for everyone…The stressful times are when people are very ill and there are arguments and there are fights, but the staff are there very quickly. It is dealt with very quickly and nipped in the bud very quickly…*

*(Female, 26–35 years, High morale acute ward)*

C. Barriers to positive interpersonal relationships and good staff morale

A number of impediments to both good staff morale and positive, engaging therapeutic relationships were identified.

i) Operational issues

The participants understood that staff had other responsibilities that could take them away from patient contact such as administrative roles (e.g. paper work, filing, electronic notes on computers), meal preparation, and observation routines. However some patients felt that these other responsibilities were excessive; that the staff were more confined to their office space and spent more time in meetings with colleagues than they should, making them less available to the patients. Lack of sufficient numbers of staff on wards was also cited as a factor for the staff not being able to meet demands of all patients. The patients described how this type of situation could lead to mounting frustration from more unwell patients and could result in aggression towards staff.

*You don’t see much of them. No. The majority of them are like, the majority of them are like in the office most of the time, you know, they’ll come out, they’ll do the medication, and they’ll go back in the office and they’ll serve us food and then go back in the office.*

*(Male, 26–35 years, High morale rehabilitation ward)*

*They do get short sometimes, many a time they get short, like not enough staff around to cater for everybody, so they’re jumping around, things like that, trying to make ends meet… you want something done, he wants something done…So you know, you might get a bit riled up.*

*(Male, 46–55 years, Low morale acute ward)*

*Very little time for patients because their taking notes all the time, doing sort of, doing meals, cleaning up, checking, sometimes they do their own bits as well.*

*(Male, 26–35 years, Low morale acute ward)*

ii) Staff lack of interest

Bank or agency nurses were used to fill in for absent staff at times but patients did not feel they benefited from this arrangement. Bank nurses mainly appeared to serve an instrumental role rather than an interpersonal role. The patients perceived the bank nurses as not as interested in their work as regular staff and as unaware of usual ward routines and procedures. This was compounded by patients feeling uncomfortable about developing rapport with a person who only worked on the ward temporarily.

*There’s no relationship there between you, so if you’ve got a problem it means you’ve got to talk to a stranger and that’s not nice. It’s much nicer to talk to somebody you’re accustomed to. Do you know what I mean? It’s…the bank staff are just… I won’t say they don’t do a good job. They do, but it’s nicer if it’s regular staff.*

*(Female, 36–45 years, High morale acute ward)*

*You become cynical about it all. You know, you operate in a way which is very narrow because that’s the way you can get through the day. Whereas I feel that, sometimes I feel that the staff could be a bit broader. It’s an intense environment, they take a narrow road, but that in turn makes the patients even more agitated.*

*(Male, 46–55 years, Low morale acute ward)*

iii) Impact of aggression and violence

Patients were very aware of deleterious consequences on staff of aggression from unwell patients. The patients felt that despite the staff having training in containing aggression, this unavoidable and unpredictable part of their job could be damaging to their emotional wellbeing. Verbal abuse as well as physical could be harmful.

*I’m sure it affects them. I’m sure it affects their feelings of themselves and their job and their self-esteem.*

*(Male, 46–55 years, Low morale acute ward)*

Like when there have been fights and people with aggressive behaviour…talking to staff like they are piece of s*** or something and they’re in their faces and calling them names and everything and trying to hit them and throw furniture around…I think that will get them down.

*(Female, 36–45 years, High morale acute ward)*

*It’s too hard for the staff and I feel sorry for them because of some of the patients…I feel terrible when the patients are after staff and they are shouting.*

*(Female, 56–65 years, High morale acute ward)*

Overall the participants placed high value on collaborative approaches to therapy and nursing care by enthusiastic staff who treated patients as ‘people’. They were also clearly aware of the impact that too many duties not directly related to patient contact, aggressive patient behaviour, and low staff numbers could have on depleting staff members’ internal resources, ultimately affecting their sense of satisfaction and enjoyment at work and their ability to engage in positive therapeutic relationships.

## Discussion

Our study adds to the findings of previous studies in three main ways: we triangulate previous findings on the determinants of staff morale by providing a patient perspective, we explore the reciprocity of the staff-patient relationship on inpatient wards, and we confirm previous findings regarding the aspects of hospital care that are most important to patients.

### Determinants of staff morale

A central theme emerging from these data was that of “enabling people, not completing tasks”. The patients described a circular relationship between good staff job satisfaction and positive relationships between patients and staff. Good morale was seen as mainly arising from positive experiences of contributing to the lives of the patients, with high morale resulting when there were good opportunities for staff to interact on a personal and positive level with patients and where teamwork and the work environment supported these endeavours. Conversely, staff morale was viewed by the patients as being under threat if barriers existed to meaningful staff-patient interactions (e.g. low staffing levels, administrative responsibilities which took staff away from the wards to offices) or if staff-patient interactions excessively depleted the staff members’ capacity to give of themselves (e.g. violent or abusive interactions that, in excess, became draining for staff). Patients found it difficult to comment on how staff morale could be improved. In their responses patients tended to describe personal experience of interaction with staff and ward life, their views about desirable staff attitudes and attributes, and their observations about factors affecting staff morale. Very similar themes to these are reported from interviews with staff carried out within the same study [15], providing some confirmation of these findings though triangulation. Team cohesion and the wider processes of the institution were understandably less prominent than in clinicians’ accounts of the pressures and rewards of their work, although some patient participants acknowledged the potential importance of these.

No clearly discernible differences were observed between patients who were on wards deemed to have high staff morale versus wards deemed to have low staff morale, either in terms the patients’ experiences of ward life, their attitudes towards staff, or their views on what constituted good or bad patient-staff interactions. Greater diversity of opinions was reported by participants within wards clustered by ‘high’ or ‘low’ morale than between these clusters.

### The reciprocity of the staff-patient relationship

A striking aspect of our data was the illumination of the reciprocal aspects of staff-patient relationships. Whereas patients are often conceptualised either as passive recipients of care or as a source of disturbance and difficulty, our interviews suggested a more complex relationship. Patients reported closely observing staff, considering the impact on them of their own and other patients’ behaviour, and on occasions seeking to care for the staff, for instance by trying to reduce demands at times when they felt staff were in difficulty. This indicates that it may be fruitful, as suggested by Halbesleber and Rathert [23] to regard patient-clinician relationships as dyadic ones to which both parties bring resources as well as demands, and through which both parties aspire to achieve positive, productive interactions.

### Aspects of inpatient care valued by patients

Our findings echo those of previous studies [9, 24] regarding the high importance placed by patients on positive interactions with staff, and on their wish for contact with them during daily life on the wards. Inpatient staff with positive personal attributes had the potential to significantly improve patients’ experiences of therapy and ward life. As in previous studies [9, 15] it also appeared that lack of staffing and excessive administrative demands are problematic because they impinge on the time available to make meaningful connections with patients.

Based on a qualitative investigation of nurse-patient interactions in acute inpatient wards, Cleary and colleagues [25] suggested that skilled nurses apply various interpersonal approaches and modalities to engage with acutely unwell patients. Patients value nurses who are humane, who use non-judgmental approaches to practice, and who exhibit patience, persistence, respect, a sense of humour, and imagination in developing effective therapeutic relationships. Sweeney and colleagues [9] similarly found that patients in acute care settings saw staff’s basic human qualities as central determinants of the quality of therapeutic alliances.

In our results, patients felt skilful communication from staff could build better therapeutic relationships and could help deal with hostility and aggression in a more humane manner, with positive impacts on both patient and on staff morale. Thus patients wanted to be seen and treated with dignity as ‘people’. Likewise, they are able to view staff as ‘people’, often observing them carefully and desiring opportunities to reciprocate in the provision of care in an ordinary human manner, i.e. looking after one another. This is congruent with the person-centred care approach, where patients are seen as people who are experts in their condition and can be empowered to participate in their own care, and staff are also seen as people who engage in relationships rather than as impersonal authority figures [26, 27].

### Strengths and limitations

This study purposively recruited patients from variety of clinical and geographical settings. The wards selected were acute, rehabilitation and intensive care units. Patients interviewed included people from a wide range of ages, ethnicities, gender and lengths of stay. All these factors may improve the relevance of the results to other inpatient mental health wards. Selection of wards in participating trusts was purposive but feasibility and convenience were also factors in recruiting them. Individual interviews were coded independently by authors with a consensus being reached on emerging themes only after several discussions. This process helped to ensure the credibility of the results.

It is possible that level of recovery, diagnosis and historical experiences may have influenced these participants’ narratives. However, an effort was made to recruit patients who were further in their recovery or were at point of discharge. It is also possible that the participants may have felt less able to voice negative views as they were interviewed within the institution in which they were receiving care. Views expressed, which were often fairly sympathetic towards ward staff, might have been different if participants had been interviewed after discharge from the wards. They might also have expressed different views if interviewed by other service users. It is also possible that some of the congruence we have noted between staff and patient views is a result of social desirability: staff may feel under some pressure to say that they would like to spend more time with patients. Thematic analysis, as conducted in this study, is often used in health research and leads to interesting insights; however it is a broad approach in which some underlying complexities and nuances are likely to be lost.

### Implication for services

From a patient perspective, as in our previous study from a staff perspective, the quality of interpersonal relationships seemed to be at the core of satisfaction with life on the wards. Evans [28] found that nurses reported spending only about 40 % of their time on direct care on inpatient wards. This may have an adverse effect on quality of patient care and job satisfaction. Thus there is potential, as in the Productive Mental Health Ward initiative which has shown some promising results in the UK, for initiatives that allow patients and staff to spend more time together to be fruitful for both patient and staff morale [29, 30]. There are also potential benefits from training staff further in communication skills to support positive patient engagement and for organisations to invest in strategies to allow more contact time between staff and patients. If successful, such strategies have potential to produce favourable results for both patient experience and staff morale.

## Conclusion

This study was conducted on seven wards as a qualitative investigation into patients’ perception of factors that influence staff morale. The results highlighted the value placed by patients on more opportunities for meaningful communication and therapeutic activities with staff. Patients value being treated as ‘people’, having opportunities to develop interpersonal relationships with staff, and to contribute to ward life. Previous research suggests staff also value and derive satisfaction from contact with patients and through their role in helping patients recover. Ward routines and procedures need to be designed in a way that allows adequate time for positive nurse-patient interaction.
